# Bone Health in Rheumatoid Arthritis: An Update

**DOI:** 10.1007/s11926-026-01230-2

**Published:** 2026-08-12

**Authors:** Faidra Laskou, Elaine Dennison

**Affiliations:** Lifecourse Epidemiology Centre, School of Human Development and Health, University of Southampton, Southampton, UK

**Keywords:** Osteoporosis, Osteopenia, Rheumatoid arthritis, Bone health

## Abstract

**Purpose of Review:**

Rheumatoid arthritis (RA) affects approximately 1% of the global population and is the most common inflammatory arthritis in adults. RA is well established as a significant risk factor for secondary osteoporosis (OP). Bone loss in RA occurs at three anatomically distinct levels: periarticular osteopenia, marginal erosions at the joint periphery, and generalised systemic OP. Understanding the complex mechanism underlying osteoclast-mediated bone loss in RA is essential for the development of targeted interventions, while identification of individuals at risk of OP and fracture remains paramount. This review will highlight recent advances in these areas.

**Recent Findings:**

The cornerstone of bone loss management and prevention in RA remains disease activity control and reduction of inflammation. In this review, we will discuss the most recent advances in understanding the mechanism of bone loss in RA, the skeletal effects of current RA and anti-OP therapies in the RA population. We also highlight areas where future research may be focused to inform best management of bone health in these patients.

**Summary:**

RA associated bone loss remains a substantial contributor to morbidity, highlighting the need for improved fracture risk stratification, optimisation of therapeutic approaches, and greater standardisation of monitoring strategies. Critical evidence gaps persist, including the absence of validated RA specific fracture risk thresholds to guide treatment decisions and the lack of prospective randomised trials comparing bone protective agents in RA associated osteoporosis. Data in men with RA remain limited, and further work is required to delineate the relative skeletal effects of biologic therapies. Additionally, optimal DXA monitoring intervals for patients with stable disease on DMARD therapy are not well defined, contributing to considerable variation in clinical practice. Addressing these unmet needs will be essential to advancing bone health management and reducing fracture burden in individuals with RA.

## Introduction

Rheumatoid arthritis (RA) affects approximately 1% of the global population and is the most common inflammatory arthritis in adults [1, 2]. RA is established as a significant risk factor for secondary OP, and previous work has demonstrated that RA increases the relative risk of hip fracture by approximately 60–80% and of pelvic and vertebral fractures by similar or greater margins, with excess risk persisting after adjustment for glucocorticoid use and physical inactivity [3]. These risks operate across all age groups and both sexes, underscoring the primacy of systemic inflammation as an independent risk factor [3]. The relationship between disease activity and OP has been further investigated in a relatively high number of studies in recent years; most reports found that in RA a higher disease activity is associated with bone loss and/or OP [4–6].

Bone loss in RA occurs at three anatomically distinct levels: periarticular osteopenia, marginal erosions at the joint periphery, and generalised systemic OP [3, 7]. The dominant molecular framework driving these changes centres on the RANK/RANKL/OPG axis, with pro-inflammatory cytokines, principally TNF-α, IL-1, IL-6, and IL-17, driving osteoclastogenesis via upregulation of RANKL on synovial fibroblasts and activated T lymphocytes [3, 7]. Concurrent Wnt/DKK-1/sclerostin-mediated suppression of osteoblast activity impairs coupled bone formation, and glucocorticoids (GCs) independently amplify bone loss through effects on calcium absorption, osteoblast differentiation, and sex hormone metabolism [8]. Understanding the complex mechanism underlying osteoclast-mediated bone loss in RA is essential for the development of targeted interventions This review will highlight recent advances in these areas.

Dual energy X-ray absorptiometry (DXA) is the diagnostic standard for assessing bone health in RA, while the FRAX^**®**^tool is a validated fracture probability tool (incorporating RA as one of twelve clinical risk factors) [9]. There appears to be a gap between optimal management of bone health in RA and current care, with recent data supporting this. For example, despite the widespread use and availability of DXA in clinical settings, a recent Australian study found that among 1,247 early RA patients, DXA was performed in only 46% within the first two years of diagnosis, while anti-osteoporotic medication was prescribed in 38% of those with BMD confirmed OP or osteopenia [10]. Younger men and women not on GCs were the most under screened groups, confirming a significant implementation gap between guideline recommendations and clinical practice, even in a well-characterised cohort. Falls risk has also been identified as a significant frequently unaddressed determinants of fracture risk in the RA population [11]. Investigation and management advances will therefore be discussed in this review.

The cornerstone of bone loss management and prevention in RA remains disease activity control and reduction of inflammation [8]. Conventional and biological DMARDs reduce inflammation and slow down joint erosion [12]; despite potent treat to target approaches in current clinical practice, it has been shown that RA patients have a two fold higher risk of fractures compared to the general population which highlights the need for more consideration of bone health in RA patients [13]. In this review, we will discuss the most recent advances in understanding the mechanism of bone loss in RA, and the skeletal effects of current RA and anti-OP therapies in the RA population.

## An Overview of the Pathogenesis of RA Related Bone loss

To place updates in understanding of the pathogenesis of RA related bone loss in context, we will begin by providing an overview of the systems and processes involved. Bone remodelling is a dynamic process of bone resorption, implemented by osteoclasts, and formation, implemented by osteoblasts, ensuring the maintenance of skeletal integrity and mineral homeostasis [14]. It is an interplay of molecular, mechanical, and cellular factors [15]. The imbalance in bone turnover results in a reduction in bone density and bone formation [15].

**Fig. 1:**
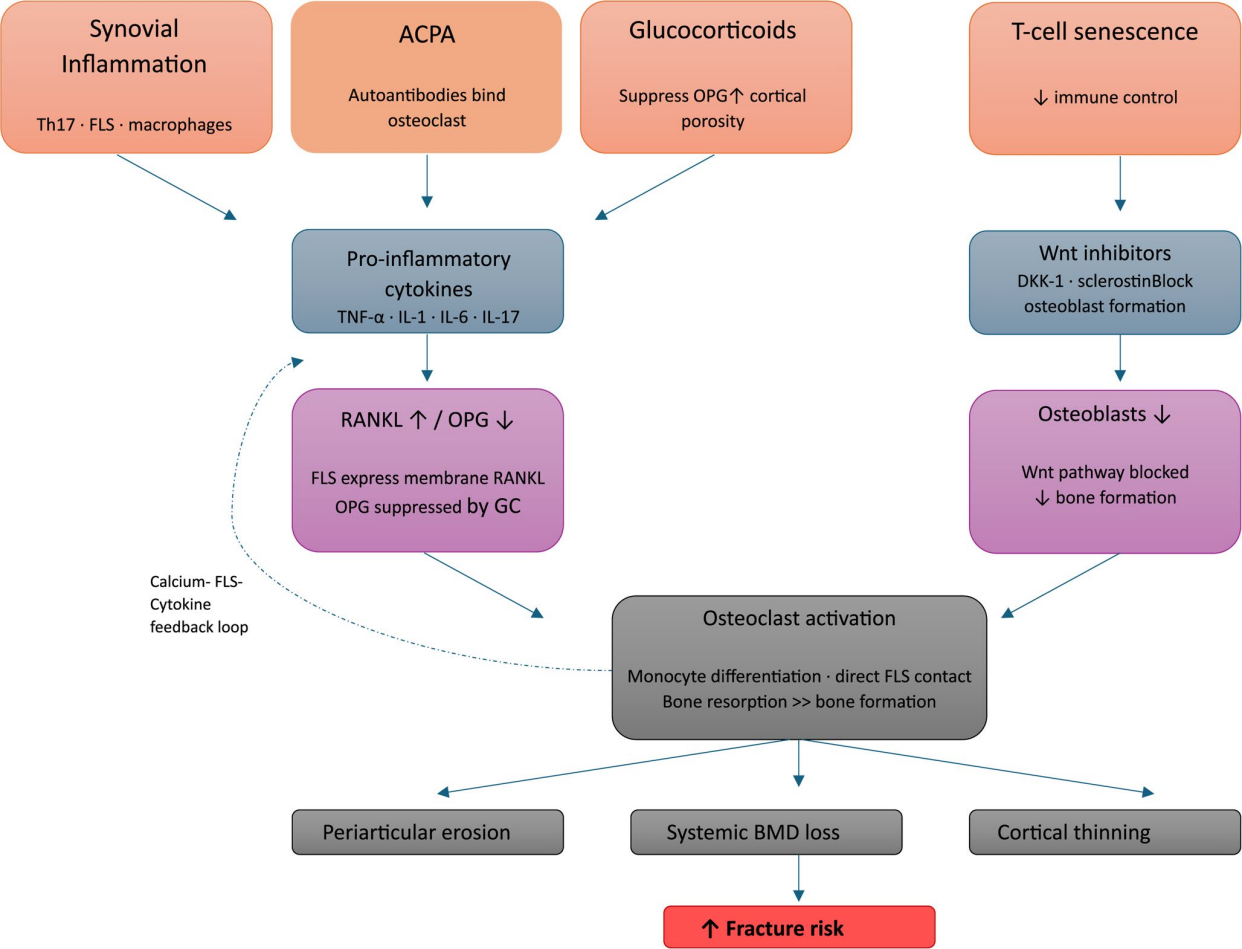
Mechanisms of osteoporosis in RA. RA:Rheumatoid arthritis, RANKL: Receptor Activator of NF-κB Ligand , OPG: Osteoprotegerin , Runx2: Runt-related transcription factor 2, Th: T helper cell; Treg: Regulatory T cell, TNF-α: Tumour Necrosis Factor-alpha, IL: Interleukin; IFN-γ: Interferon-gamma, BMP: BMP: Bone Morphogenetic Protein , DKK: Dickkopf-1 (Wnt antagonist)

Bone loss in RA arises from a complex interplay of immune-mediated, molecular, and iatrogenic mechanisms operating at both local and systemic levels, as shown in Fig. 1. Recent advances have introduced new factors into our understanding of the classic route by which osteoclast activation is related to the RANK/RANKL/OPG pathway; the inflammatory state mediated by IL-1, IL-6, IL-8, and TNF-α activates and differentiates osteoclasts not only through RANKL, but also through the Wnt/DKK1/sclerostin pathway, leading to bone loss [16]. Pro-inflammatory cytokines including IL-6 and its soluble receptor, induce RANKL expression in fibroblast-like synoviocytes (FLS), and are vital for TNF-α and IL-17 mediated RANKL upregulation, collectively driving osteoclast differentiation and periarticular bone erosion [7]. Th17 cells, abundant in the inflamed synovium, amplify inflammatory cytokine secretion, trigger RANKL expression, and stimulate synovial fibroblasts via IL-17 to further upregulate RANKL, while circulating monocytes are transformed into osteoclasts under the influence of these pro-inflammatory signals; TNF additionally suppresses osteoblast-driven bone formation through inhibition of Wnt signalling via DKK1 and sclerostin, further restraining bone regeneration [7, 17]. Synovial fibroblasts function as “RANKL factories,” expressing exceptionally high levels of membrane-bound RANKL that efficiently activate osteoclast precursors via direct contact, while synovial fibroblasts, osteocytes, and M1 macrophages overexpress the Wnt inhibitors DKK-1 and sclerostin, blocking Wnt ligand interactions and inactivating key osteogenic transcription factors [18].

Autoantibodies, particularly anti-citrullinated protein antibodies (ACPA), are also involved in the pathogenesis of bone loss; ACPA can induce osteoclast differentiation and activation even before arthritis onset, with myeloid precursors expressing citrullinated vimentin on their surface that serves as a target for ACPA, which bind Fc-gamma receptors and promote CXCL8-driven osteoclast proliferation and maturation [16]. Th cell senescence, with a presumed disability to control the systemic immune and osteoclastogenic status, may play an important role, resulting in increased osteoclast activity, decreased osteoblast function, and deterioration of bone metabolic and mechanical properties [3].

Glucocorticoids have historically been widely used to manage RA disease activity but may compound this skeletal burden by downregulating bone formation and upregulating bone resorption which can indirectly affect calcium metabolism contributing to secondary hyperparathyroidism [19]. Prolonged glucocorticoid exposure suppresses osteoprotegerin (OPG) expression in osteocyte-enriched cortical bone without necessarily increasing RANKL, shifting the RANKL/OPG balance in favour of bone resorption and increasing osteoclast numbers at the endocortical surface, reducing cortical thickness and increasing porosity in mice [19, 20].

Together, these converging pathways, cytokine-driven RANKL/OPG imbalance, impaired Wnt-mediated bone formation, ACPA-mediated osteoclastogenesis, immune cell senescence, and glucocorticoid-induced cortical bone loss, account for the substantially elevated fracture risk observed in patients with RA [21].

## Updates in Pathogenesis

A better understanding of pathogenesis in RA related bone loss affords greater insights into management of the condition, and an appreciation of possible therapeutic targets (Fig. 1). Beyond the established RANKL pathway, several other mechanisms have been highlighted recently. These are described below.

Synovial fibroblasts include heterogeneous subsets of both pro-inflammatory and tissue-destructive cell types. Studies on synovial tissues have found that fibroblast-like synoviocytes (FLS) are RANKL and pro-inflammatory cytokine expressing and producing cells in patients with RA [22]. Anti-citrullinated protein antibody increased RANKL and pro-inflammatory cytokine expression in RA-FLSs, and it has been suggested that ACPA-activated RA-FLSs could augment osteoclastogenesis supporting a novel pathological role of ACPA in RA pathogenesis. In addition, it has been recently suggested that blocking the CD64- and PAD-2-mediated pathways may ameliorate ACPA mediated joint destruction in patients with ACPA positive RA [22]. Recent studies on synovial fluid have also demonstrated that calcium released from bone by osteoclasts activates FLS migration and cytokine production, and that cytokine-stimulated FLS medium promotes further osteoclastogenesis, a self-amplifying destructive loop. This suggests that modulation of this crosstalk by inhibiting the calcium and cytokine feedback loop could be used to develop pioneering strategies [23].

In RA it has been suggested that several alterations in systemic cytokine titres can be detected, which may influence bone remodelling and, consequently, the risk of osteoporotic fractures [3]. This suggestion was supported in a recent study where serum levels of TNF-α, IL-6, and IL-17 were higher and levels of IL-4 and IL-10 were lower in RA patients when compared to those without OP. In the same study it was also suggested that a combination of DAS28 (disease activity score), and quantification of IL-4, IL-10, and IL-17 levels could predict the incidence of OP in RA [4]. IL-8, a chemokine that attracts neutrophils and other leukocytes to sites of inflammation, has been shown to be able to activate and differentiate osteoclasts through the wnt/DKK1/sclerostin pathway leading to bone loss [24].

In other work sited in a prospective cohort of early RA patients, researchers found that high baseline circulating Th17 cell proportions predicted trabecular bone loss over 48 weeks as measured by HRpQCT, while high Treg proportions were protective, directly linking the Th17/Treg ratio to skeletal outcomes [25]. Dickkopf-1 (DKK-1) and sclerostin, both elevated in RA synovium, suppress canonical Wnt signalling and reduce osteoblast number and activity, accounting for the decoupling of resorption from formation that characterises RA bone loss. In recent work, serum DKK-1 levels have been shown to correlate with reduced TBS in RA patients, providing a link between Wnt pathway suppression and trabecular microarchitectural deterioration that is not captured by BMD alone [13]. The therapeutic relevance of this pathway is now demonstrable, with romosozumab, a monoclonal antibody targeting sclerostin, showing activity both anabolically (promoting bone formation) and antiresorptively (reducing bone resorption) [26].

In other work, bone resorption markers were reported to be significantly elevated in an RA patient population compared with osteoarthritis patients and healthy controls, with a pattern consistent with uncoupled, resorption-dominant remodelling, with a particularly strong effect on bone formation [24]. These findings may allow identification of different subtypes of bone pathology in RA. In a cross-sectional cohort of 283 RA patients, the composite bone turnover markers (BTM) model (P1NP, CTX-I, and osteocalcin, combined with clinical variables) achieved an AUC of 0.81 (95% CI 0.75–0.87) for identifying DXA-confirmed OP, substantially outperforming individual markers [27].

Genetic studies have also advanced our understanding of the pathogenesis of bone loss in RA. Researchers used Weighted Gene Co-expression Network Analysis (WGCNA) and machine learning to identify two key apoptosis-linked genes, ATXN2L and MMP14, as shared between RA and OP, suggesting potential therapeutic targets for future research, although more research is needed [28]. A separate Mendelian randomisation study suggested a causal relationship between genetically predicted RA liability and fracture risk, lending additional support to the suggestion that RA is a truly independent skeletal risk factor rather than an epiphenomenon of GC use or immobility [29]. In another Mendelian randomisation study, transcriptome wide analysis identified 23 shared genes between OP and RA providing a link through biological pleiotropy. These findings further support the notion that BMD decline might be a preclinical phenomenon rather a clinical outcome in the pathogenesis of RA [30].

## Fracture Incidence in RA - Recent Evidence

The clinical outcome related to RA related bone loss is fracture. A 13-year prospective analysis of the Institute of Rheumatology Rheumatoid Arthritis (IORRA) cohort investigated fracture incidence in a large Japanese RA cohort and found that fracture rates increased significantly over the observation period despite DMARD therapy [31]; the incidence of all and non-vertebral fractures increased from 47.2 to 36.7/1000 person-years in 2011 to 52.8 and 43.0/1000 person-years in 2023, respectively. The mean age of participants in this study was 58.4 years while 83.9% were women, with an average of 11.5 years disease duration. These observations suggest that structural contributors to fracture risk such as cumulative bone loss, trabecular microarchitectural damage, and medication effects, may continue to worsen even as synovitis is controlled [31]. These data underscore a view that effective RA treatment is necessary but maybe not wholly sufficient for fracture prevention [8, 31].

Recent epidemiological studies have highlighted high fracture risk in patients with RA, adding to previous literature. A recent Spanish study highlighted the high fracture risk in patients with RA showing that the rates of major osteoporotic fracture in post-menopausal women with RA (with a median age of 64 years) were 3.55/100 compared to matched controls with a rate of 0.72/100. More than 60% of these fractures occurred in the spine. Those patients with a previous fragility fracture have the highest risk of further fracture [32]. In other work based in the Nord Trondelag Health study (HUNT), researchers reported that participants with RA had an HR of 1.41 (95% CI 1.13 to 1.74) of fractures compared with those without RA [33]. Finally in a real world DXA cohort comparing RA to healthy controls and a non RA cohort with a major osteoporotic fracture risk, almost one in five RA patients and more than one in four of an at-risk group had a prevalent MOF; those with prior MOF had significantly lower BMD at each measured site. Increased number of fractures were reported in RA patients despite having similar age and bone mineral density measurements to healthy controls [34].

## RA Treatment and BMD

As RA treatment has evolved in recent years to include biologic therapies, one might anticipate that this may link to better bone health and a recent long term cohort study report has supported this assumption [35]. However, the evidence remains somewhat conflicting [36, 37]. In a recent systematic review, use of any DMARD appeared to be beneficial towards BMD; bDMARD and tsDMARD appear to have a greater beneficial effect on BMD but further research in required to understand the role of specific DMARDs on bone [37] as included studies were deemed high risk of bias, although most were prospective or randomised controlled in design [37].

Current research highlights potential differences between classes of DMARDs that requires further research [37]. For example, in a recent retrospective study, using a claim database, use of TNF-a and IL-6 inhibitors in RA management was associated with apparently better BMD preservation, while TNF-a inhibitors may also offer advantages in all-cause mortality warranting further investigation [38]. Despite these observations, the duration of b/tsDMARD use and activity score were not associated with BMD maintenance in a multicentre observational study, suggesting that even in disease remission, BMD is still decreased without concomitant OP medications, highlighting the need for identifying individuals at risk of OP, independently of disease activity [36].

JAK inhibitors (JAKi), now well-established in RA management, signal through the JAK-STAT pathway and suppress a broad range of cytokines driving both inflammation and osteoclastogenesis [39]. Recent experimental data have begun to delineate the site- and context-specific skeletal effects of JAKi [39]. In collagen-induced arthritis mouse models, JAKi were found to suppress osteoclastogenesis at sites of joint erosion, while at periarticular and systemic bone sites they additionally promoted osteoblastic bone formation, suggesting a dual mechanism that may be more bone-protective than simple anti-inflammatory suppression alone [39]. In recent human studies, BMD measurement changes in RA patients treated with baricitinib, a JAK1/2 inhibitor, over 12–24 months were compared with historical controls and matched comparators receiving conventional DMARDs; baricitinib-responders showed stable BMD while BMD decrease was noted to Baricitinib nonrespondents [40]. While these data remain observational and await prospective trial confirmation, they raise the clinically important possibility that choice of DMARD may itself influence fracture risk beyond its effect on disease activity.

Finally, steroid use remains necessary in some RA patients despite therapeutic advances. While steroids are known to be detrimental to bone health at higher doses, a recent systematic review evaluated the effects of two years low dose glucocorticoids in RA patients (< 7.5 mg/day), on BMD and fracture risk, concluding that even low dose steroids could lead to bone loss at the lumbar spine, but not at the femur [41].

## RA Treatment and Fracture Risk

The clinical outcome of low BMD is fracture, but fewer studies that include fracture data are available. One example of recent available data comes from the HUNT study, where the hazard ratio of fractures in bDMARD users compared with participants without RA remained largely similar to the main results, while csDMARD users had a comparable fracture risk with participants with RA overall, indicating that anti-inflammatory treatment and/or antiosteoporotic treatment are important for fracture prevention in this patient group [33].

## Nutrition and Muscle Health in RA

Patients with RA are prone to develop malnutrition as their medication often affects appetite, nutrient absorption, digestion, and increased nutritional needs due to inflammation, making them at nutritional risk, which in turn impacts musculoskeletal health [42]. A recent clinical study specifically examined malnutrition and osteosarcopenia in elderly women with RA, finding that nutritional deficit and low muscle mass were independently associated with low BMD and increased fracture risk in this population [43]. These findings are clinically relevant because sarcopenia and malnutrition are often under-recognised and potentially modifiable contributors to bone fragility in RA, particularly in older women.

## Risk Fracture Assessment

### FRAX and RA

FRAX remains the most widely used fracture probability tool globally, and RA is one of only twelve clinical risk factors explicitly embedded in its algorithm [44]. However, several limitations specific to RA have been highlighted in recent literature. FRAX captures the binary presence of RA as a risk factor but does not account for disease severity, disease activity, duration, or degree of functional impairment, all of which modulate fracture risk [44, 45]. It is notable that a recent meta-analysis demonstrated that a diagnosis of RA alone confers an increased risk of any clinical fracture varying between 49 and 93% independent of BMD, age, sex and exposure to glucocorticoids; the impact of DMARD use and type of DMARD was not accounted for in the algorithm [45]. In addition FRAX does not separately accommodate glucocorticoid dose in a continuous fashion; users must adjust manually for high-dose GC use [46].

Validation of FRAX in modern RA patients has previously been lacking and this was addressed in a recent study that suggested that FRAX continues to be an appropriate tool for fracture risk assessment in RA patients though a consideration remains as to whether FRAX might overestimate risk in those already at high predicted risk [47]. Further validation is now required in younger population with greater biologic exposure [47].

Performance of FRAX in different ethnicities was evaluated in a recent study using US-FRAX in a multiethnic cohort of US women with RA [48]. FRAX performed well for short-term hip and major osteoporotic fracture prediction overall, but calibration was less reliable in Black, or African American and Hispanic and Latina women, where the tool systematically underestimated fracture risk [48]. These findings have important implications for RA bone health management in diverse populations and support the need for fracture risk models that balance inclusivity and individual levels precision [48].

### Prediction Models

Machine learning (a branch of artificial intelligence (AI)) offers another opportunity to improve fracture risk recognition. Despite major advances in RA therapy, including effective use of synthetic and biologic DMARDs, their impact on preventing bone mineral density loss remains limited [31, 37]. Early OP screening is further hindered by delays in routine practice [10]. Clinical prediction models (CPMs) offer a potential solution by enabling early risk stratification and targeted intervention [27, 49]. Although several models exist, many are currently limited by small cohorts, insufficient external validation, and challenges in clinical applicability [27].

## Updates in Management

### Bisphosphonates and Denosumab in RA-Associated Osteoporosis

Bisphosphonates (BPs) remain the most widely prescribed antiresorptive therapies for OP in RA [50, 51]. Their efficacy for vertebral, non-vertebral, and hip fracture reduction in the general OP population is well established; however, dedicated RCT data in RA patients specifically remain limited [52]. RA patients are primarily included in Glucocorticoid-Induced Osteoporosis (GIOP) trials, therefore providing some indirect evidence of fracture reduction [53]. For example in a double blind placebo controlled study focusing on the effects of oral BPs in RA patients treated with low dose steroids, a significant increase in BMD was found 1 year after starting treatment but no difference in fracture risk reduction was observed; this study remains the only study focusing on BPs and fracture risk in RA [54]. This was also supported by a recent prospective cohort that reported that BPs are protective against bone loss even over a short period [55]. However, studies on bone biopsies from RA patients on long term BP therapy support the concept that other treatment options might be required in GIOP [56]; GIOP patients receiving long term BP therapy had lower bone material strength, secondary to a hypomineralised bone matrix, compared to age matched postmenopausal women, indicating that BPs alone might not sufficiently reduce the risk of GIOP fragility fractures [56].

Denosumab, the fully human monoclonal antibody against RANKL, has particular mechanistic appeal in RA given the centrality of the RANK/RANKL axis to RA-mediated osteoclastogenesis [57]. A recent meta-analysis of head to head trials comparing BPs to denosumab in RA showed that denosumab improved BMD significantly more than BPs at 12 and 24 months with only one study showing greater osteoporotic fracture reduction with denosumab [58]. This suggests that in patients treated with BPs, switching to denosumab could result in greater BMD increase compared to switching to another BP [58]. Another recent meta-analysis of all studies supported the evidence that denosumab increases BMD, but this might be influenced by the age and sex of the RA patient [59].

A 2024 narrative review of 37 studies reported superior BMD improvements with denosumab over BPs and demonstrated additional benefits including improvements in appendicular lean mass and grip strength, clinically relevant in RA patients prone to sarcopenia [60]. A key caveat with denosumab is the rebound phenomenon upon discontinuation: rapid increases in bone resorption markers and accelerated BMD loss, occasionally resulting in multiple vertebral fractures, can occur without prompt transition to an antiresorptive agent [61]. Sequential therapy planning must therefore be embedded in any denosumab prescribing decision. denosumab is therefore often most appropriately used in older people where it can be continued lifelong (e.g., 10 years or more, without a strict requirement to stop at 10 years).

### Romosozumab in RA Associated Osteoporosis: New Evidence

Romosozumab, the anti-sclerostin monoclonal antibody administered as 210 mg subcutaneously monthly for 12 months, has a dual mechanism, stimulating bone formation and inhibiting bone resorption simultaneously, and produces markedly greater BMD gains than antiresorptives alone, particularly at the lumbar spine [62].

In a retrospective study of 171 postmenopausal patients (made up of 59 in an RA group and 121 in a non-RA group, no glucocorticoids) receiving uninterrupted romosozumab, the rate of increase in P1NP (a bone formation marker), and decline in TRACP-5b (a bone resorption marker), exhibited smaller changes in the RA group, while BMD increase after 12 months was lower in the RA patients, indicating that the efficacy of romosozumab may be attenuated in RA [63]. The comparable clinical efficacy of romosozumab to denosumab in RA patients receiving GCs was tested in a randomised study in which romosozumab produced significantly larger lumbar spine BMD gains at 3 months than denosumab in RA patients with severe OP [64].

A cardiovascular safety signal associated with romosozumab warrants consideration however; romosozumab requires risk assessment in patients with prior myocardial infarction or stroke [65]. This is important as RA patients have an elevated cardiovascular risk, making pre-prescribing cardiovascular evaluation mandatory [66].

### Teriparatide and Abaloparatide

No studies of the effectiveness of teriparatide or abaloparatide in RA patients specifically are available; The clinical effectiveness of teriparatide has been investigated compared to risedronate in GIOP male patients (22.7% of whom were receiving GC due to RA) where larger improvements in spinal BMD, microstructure, and FE-derived strength were demonstrated in the teriparatide treated group compared to risedronate [67]. Additionally, BMD increased more in patients receiving teriparatide than in those receiving alendronate in GIOP in a separate randomised double blind controlled study; almost 52% and 46% of patients had a diagnosis of RA on GCs in the study who were randomised to receive alendronate or teriparatide respectively [68].

Recent evidence of osteoporosis management in RA is summarised in Table 1.

**Table 1:** Summary of pharmacological therapies for RA-associated osteoporosis; recent evidence

Drug	Study design	Population	BMD outcome	Fracture outcome	Comments
Bisphosphonates	Double-blind RCT	RA patients on low-dose GCs	Significant ↑ BMD at 1 year	No significant reduction in fracture risk	Only RCT reporting fracture outcomes specifically in RA
	Bone biopsy studies (long-term BP therapy)	GIOP patients vs. age-matched postmenopausal women	Lower bone material strength; hypomineralised matrix	Not reported	Suggests BPs alone may be insufficient for GIOP fracture prevention
Denosumab	Meta-analysis (head-to-head RCTs vs. BPs)	RA patients	Significantly greater BMD gains at 12 and 24 months vs. BPs	One included study demonstrated greater fracture reduction vs. BPs	Fracture reduction data limited to single study within meta-analysis
	Meta-analysis (all studies)	RA patients; effect modified by age and sex	Consistent ↑ BMD across studies	Not pooled	Heterogeneity in patient demographics may limit generalisability
	Narrative review (37 studies)	RA patients	Superior BMD gains vs. BPs; ↑ appendicular lean mass and grip strength	Not reported	Rebound resorption and vertebral fracture risk on discontinuation; sequential antiresorptive therapy required
Romosozumab	Retrospective cohort	Postmenopausal women: RA (*n* = 59) vs. non-RA (*n* = 121), GC-free	Smaller ↑ P1NP and smaller ↓ TRACP-5b in RA; lower BMD gains at 12 months vs. non-RA	Not reported	Suggests attenuated efficacy in RA; retrospective design
	Randomised study	RA patients with severe OP receiving GCs	Greater lumbar spine BMD gains at 3 months vs. denosumab	Not reported	Cardiovascular safety signal (ARCH trial); mandatory CV risk assessment required prior to prescribing given elevated CV risk in RA
Teriparatide / Abaloparatide	RCT (GIOP)	Male GIOP patients (22.7% RA); teriparatide vs. risedronate	Greater ↑ spinal BMD, microstructure, and FE-derived bone strength with teriparatide	Not reported	No RA-specific RCT; evidence derived from GIOP subgroup
	Randomised double-blind RCT (GIOP)	GIOP patients (~ 52% RA in alendronate arm; ~46% in teriparatide arm); teriparatide vs. alendronate	Greater BMD gains with teriparatide vs. alendronate	Not reported	RA patients not analysed separately; no data available for abaloparatide in RA or GIOP

*RCT* randomised controlled trial, *RA* Rheumatoid arthritis, *GIOP* Glucocorticoid induced osteoporosis, *BMD* Bone mineral density, *BP* Bisphosphonates

### Current and Updated Guidelines

There are no guidelines that specifically address OP treatment for patients with RA; if individual risk factors suggest the needs for formal bone health assessment, completing a risk assessment using FRAX, incorporating BMD obtained by DXA scan when available is recommended. The 2022 ACR Guideline for the Prevention and Treatment of GIOP represents the most comprehensive recent framework for bone protection in RA patients receiving GCs [69]. It is also recommended that clinicians use the NOGG recommendations to guide further actions [9].

## Conclusion and Future Directions

Several important unresolved areas should guide future research (Table 2). First, there are no validated RA-specific fracture risk thresholds for treatment initiation, and existing tools (FRAX, Trabecular bone score) were not derived in RA populations. Second, prospective RCT data comparing bone-protective agents specifically in RA-associated OP remain sparse; most evidence is extrapolated from primary or glucocorticoid induced OP trials. Third, men with RA remain under screened and undertreated, and dedicated RCT data in men, including those with RA, are needed to inform evidence-based thresholds. Fourth, the differential bone-protective effects of JAKi versus biologics need confirmation in adequately powered prospective studies with BMD and fracture endpoints. Fifth, optimal monitoring intervals for DXA in RA on stable DMARD therapy require prospective study, as current practice varies widely. Finally, the possibility of integration of bone biomarkers (CTX-I, P1NP, bone-specific ALP) into routine RA monitoring to guide treatment decision-making deserves prospective evaluation.

**Table 2:** Unmet needs and future research directions in RA associated osteoporosis

	Unmet Need	Future Direction
1	No validated RA-specific fracture risk thresholds for treatment initiation	Develop RA-specific thresholds; existing tools (FRAX, TBS) were not derived in RA populations
2	Sparse prospective RCT data on bone-protective agents specifically in RA-associated osteoporosis	Conduct RCTs in RA-OP populations rather than extrapolating from primary or glucocorticoid-induced OP trials
3	Men with RA are under-screened and undertreated	Dedicated RCT data in men (including those with RA) to inform evidence-based screening/treatment thresholds
4	Unclear differential bone-protective effects of JAK inhibitors vs. biologics	Adequately powered prospective studies with BMD and fracture endpoints to confirm differences
5	No established optimal DXA monitoring intervals for RA patients on stable DMARD therapy	Prospective studies to standardize monitoring intervals, given current wide variation in practice
6	Bone biomarkers (CTX-I, P1NP, bone-specific ALP) not yet integrated into routine RA monitoring	Prospective evaluation of biomarker integration to guide treatment decision-making

## Key References

- Aritomi T, Sonomoto K, Nakayamada S, Tanaka H, Nagayasu A, Kubo S, et al. Biologic andTargeted Synthetic Disease-Modifying Antirheumatic Drugs Do Not Arrest Bone Loss inPatients With Rheumatoid Arthritis: A Long-Term Multicenter Observational Study.Arthritis Rheumatol. 2026. (36)
  - ○ This long term multicentre observational study demonstrates that biologic and targeted synthetic DMARDs, despite effectively controlling inflammation, do not halt progressive bone loss in RA. Its importance lies in underscoring the need for dedicated osteoporosis prevention strategies even in patients receiving advanced RA therapies.
- Furuya T, Inoue E, Yamanaka H, Harigai M, Tanaka E. Increasing fracture incidence over 13 years in patients with rheumatoid arthritis from the IORRA cohort. J Bone Miner Metab. 2025;43(4):411–8.(31)
  - ○ Using 13 years of IORRA cohort data, the authors show a steady increase in fracture incidence among RA patients despite therapeutic advances. This study is important because it highlights a persistent and growing unmet need in fracture prevention within modern RA care
- Kanis JA, Johansson H, McCloskey EV, Liu E, Schini M, Vandenput L, et al. Rheumatoid arthritis and subsequent fracture risk: an individual person meta-analysis to update FRAX. Osteoporos Int. 2025;36(4):653–71.(45)
  - ○ This individual level meta analysis confirms that RA confers a significant independent increase in fracture risk, prompting an update to the FRAX algorithm. Its importance lies in improving global fracture risk prediction accuracy, directly influencing clinical decision making.
- Lyon A, Lester S, Stanhope J, Lynch T, Black R, Barrett C, et al. Dual-energy X-ray absorptiometry and anti-osteoporotic medication use in Australian patients with early rheumatoid arthritis using data from the Australian Rheumatology Association Database. Intern Med J. 2025;55(7):1127–35.(10)
  - ○ Analysis of the Australian Rheumatology Association Database reveals that while many early RA patients undergo DXA and receive anti osteoporotic therapy, substantial gaps in assessment and treatment persist. This study is important because it identifies early missed opportunities for fracture prevention in routine practice despite well recognised pathways for OP identifiaction.
- Schulz N, Asendorf T, van Wijnen P, Wilhelmi T, Müller-Ladner U, Lange U, et al. Bone mineral density during treatment with the Janus kinase inhibitor baricitinib in patients with rheumatoid arthritis: A monocentric observational study. Calcif Tissue Int. 2025;116(1):101.(40)
  - ○ This monocentric observational study reports that baricitinib therapy is associated with stable or slightly improved BMD in RA patients over time. Its importance lies in providing emerging real world evidence that JAK inhibitors may have a neutral or favourable skeletal profile.
- Taylor-Williams O, Godwin R, Carvallio R, Taylor-Williams M, Barrett C, Inderjeeth C. Bone mineral density in rheumatoid arthritis patients on antirheumatic therapies: a systematic review. Bone. 2026 May;206:117812. doi: 10.1016/j.bone.2026.117812. Epub 2026 Jan 28. PMID: 41616894.(37)
  - ○ This systematic review reports that cs, b-, and /or ts-DMARDs therapy is associated with stable or slightly improved BMD in RA patients over time. Its importance is in synthesising the evidence base and highlighting the lack of definitive conclusions, reinforcing the need for targeted bone specific interventions in RA.
- Vera-Ponce VJ, Ballena-Caicedo J, Zuzunaga-Montoya FE, Loayza-Castro JA, Valladolid-Sandoval LAM, Vásquez-Romero LEM, et al. Prevalence of osteoporosis in chronic diseases: an umbrella review of 283 observational studies from 13 systematic reviews. BMC Rheumatol. 2025;9(1):66.(2)
  - ○ This umbrella review of 283 studies shows that osteoporosis is highly prevalent across chronic diseases, including RA. Its importance lies in quantifying the systemic burden of osteoporosis and emphasising the need for proactive bone health management in chronic inflammatory conditions

## Data Availability

No datasets were generated or analysed during the current study.
